# Wrecked Clergymen

**Published:** 1877-09

**Authors:** 


**WRECKED CLERGYMEN.**—The utter stupidity of the men who have the control and management of Theological Seminaries, is inconceivable, and the young men who go there do, in the judgment of charity, possess more piety than brains; their zeal is not according to knowledge; they cheat themselves, they cheat their parents, and they cheat the church. A considerable part of the money which is expended in paying professors' salaries and in sustaining "poor and pious young men," comes from people who have to work for a living; from farmers, mechanics; from girls and old women who make it by sewing and knitting; from persons who, by severe economies, lay up a little as a matter of duty and of love to the Master. As faithful stewards, the men who distribute this money, or who order its distribution, are bound to do it wisely, to the best advantage. If this is done by throwing out a bran-new preacher on the community who has barely strength to stand up long enough to read a sermon, who is so wrecked in brain and ruined in body, that it is problematical whether either will last six months from the day of his leaving the seminary—then we do not know what a faithful stewardship is. A professor in a theological seminary has no sense, never had any, never will have, beyond what he gathers in chewing Hebrew roots and ferretting out Greek themes; his whole soul is taken up in proving doctrines that never existed except in his own imagination; in showing what can not be seen; in fabrications which a breath of truth will smash to atoms. And looking forward to the time when they shall be quoted as very giants in Scriptural learning, they seem wholly to forget that the young men under their care have something else to do besides cramming their brains with theological abstractions. It really admits of debate, whether it would not be better for the world if the engine of Time had not better take a turn back by dumping every theological seminary, every medical college, and every public school into Symmes' north hole, and make another start in the direction of a better progress. Meanwhile, let this young man who wants an education, work by day for his board and tuition, and study at night by the light of a pine-knot or a bit of a rag dipped in a saucer of hog's fat.

Let the other youngster who wants to become a doctor, take up the old-fashioned plan of compounding his master's medicines, going round with him to visit patients, and gradually learn to take his place when emergencies present themselves, thus making actual observation the foundation of his study and his skill. And as to theological training, certain it is that some of our greatest divines, the men who have signalized themselves by splendid utilities, were those who plodded while they plowed, who "studied" with their own ministers, and thus literally graduated from "the school of the prophets," without being hurried to death to keep up in the class with those who had brains and nothing else.

